# Supplementary material for: Risk of Benign and Malignant Thyroid Disorders in Subjects Treated for Paediatric/Adolescent Neoplasia: Role of Morphological and Functional Screening
Source: Children (Basel). 2021 Aug 31;8(9):767. doi: 10.3390/children8090767 (PMC8468117; doi:10.3390/children8090767)
Supplement: Supplementary file 1 [file children-08-00767-s001.zip › children-1339790-supplementary.pdf]

Supplementary Table S1. The impact of gender in CHE+RTE and CHE group

	All			CHE+RTE			CHE		
	**Female n=167 (%)**	**Male n=176 (%)**	**p**	**Female n=82 (%)**	**Male n=83 (%)**	**p**	**Female n=85 (%)**	**Male n=93 (%)**	**p**
**Normal**	83 (49.7)	114	0.006	26	36	0.15	57	78	0.01
**Hypothyroidism**	30 (17.9)	24	0.31	17	17	1.0	13	7	0.15
**Hyperthyroidism**	3 (1.8)	1	--	2	1	--	1	0	--
**Chronic Thyroiditis**	4 (2.4)	4	1.0	2	2	1.0	2	2	1.0
**Thyroid nodules**	31 (18.6)	32	1.0	21	28	0.31	10	4	0.09
**Thyroid cancer**	23 (13.8)	7	0.002	17	4	0.002	6	3	0.31
